# Nonlinear Noise Cleaning in Gravitational-Wave Detectors With Convolutional Neural Networks

**DOI:** 10.3389/frai.2022.811563

**Published:** 2022-03-17

**Authors:** Hang Yu, Rana X. Adhikari

**Affiliations:** ^1^TAPIR, Walter Burke Institute for Theoretical Physics, MC 350-17, California Institute of Technology, Pasadena, CA, United States; ^2^LIGO Laboratory, MC 100-36, California Institute of Technology, Pasadena, CA, United States

**Keywords:** gravitational-wave detectors, Advanced LIGO, noise regression, machine learning, neural networks

## Abstract

Currently, the sub-60 Hz sensitivity of gravitational-wave (GW) detectors like Advanced LIGO (aLIGO) is limited by the control noises from auxiliary degrees of freedom which nonlinearly couple to the main GW readout. One promising way to tackle this challenge is to perform nonlinear noise mitigation using convolutional neural networks (CNNs), which we examine in detail in this study. In many cases, the noise coupling is bilinear and can be viewed as a few fast channels' outputs modulated by some slow channels. We show that we can utilize this knowledge of the physical system and adopt an explicit “slow×fast” structure in the design of the CNN to enhance its performance of noise subtraction. We then examine the requirements in the signal-to-noise ratio (SNR) in both the target channel (i.e., the main GW readout) and in the auxiliary sensors in order to reduce the noise by at least a factor of a few. In the case of limited SNR in the target channel, we further demonstrate that the CNN can still reach a good performance if we use curriculum learning techniques, which in reality can be achieved by combining data from quiet times and those from periods with active noise injections.

## 1. Introduction

Since September 14, 2015 (Abbott et al., 2016), gravitational-wave (GW) observatories including Advanced LIGO (aLIGO; LIGO Scientific Collaboration, 2015), Advanced Virgo (Acernese et al., 2015), and KAGRA (Kagra Collaboration, 2019) have achieved great success with dozens of GW event detected so far (Abbott et al., 2019a, 2020).

While the high-frequency (≳60Hz) part of aLIGO's sensitivity is steadily approaching its designed target especially with the implementation of quantum squeezing (Tse and et al., 2019), there is nonetheless a big gap between the current and the designed sensitivity at lower frequencies (Martynov et al., 2016; Buikema et al., 2020).

If we can remove the excess contamination in the sub-60 Hz band, it would greatly promote a wide array of science cases including the early warning of binary neutron star mergers (Cannon et al., 2012; Abbott et al., 2019b; Chu et al., 2020; Sachdev et al., 2020; Yu et al., 2021), the detection of intermediate-mass black holes (Mandel et al., 2008; Graff et al., 2015; Veitch et al., 2015; LIGO Scientific Collaboration and Virgo Collaboration, 2020), the constraining of eccentricities of binary black holes and hence their formation channels (Abbott et al., 2019; Romero-Shaw et al., 2019), and many more. Refer also the discussions in, e.g., Yu et al. (2018) and references therein.

The limiting factors to the current sensitivity below 60 Hz are “technical noises” due to environmental perturbations and control noises of auxiliary degrees of freedom. Unlike fundamental noises due to quantum and thermal fluctuations in the main GW readout channel that cannot be mitigated without instrumental upgrades, the technical noises can in principle be removed *via* regression techniques as their source fluctuations are also witnessed and recorded by hundreds of auxiliary sensors (i.e., sensors that do not detect GW signals) employed by aLIGO. In fact, the linear component of technical noises has successfully been removed in aLIGO (refer to, e.g., Davis et al., 2019; Driggers et al., 2019).

The remaining challenge of the regression problem is to tackle noises that couple to the main GW readout *nonlinearly*. In fact, many noise sources in aLIGO couple in a bilinear way to the GW readout. It happens naturally when the coupling coefficient of an auxiliary channel is modulated by some slow motion (≲1Hz) in the interferometer. The modulation destroys the linear coherence between the auxiliary channel and the GW readout, forbidding the use of a standard linear regression technique like the one employed in Driggers et al. (2019). Moreover, it is typically challenging to reconstruct the modulation directly because of the pollution from large ambient motion and cross-couplings from complicated control feedback loops stabilizing aLIGO at below 1 Hz.

Fortunately, machine learning (ML) techniques, especially the use of convolutional neural networks (CNNs; Lecun et al., 2015), offer an attractive potential solution to the nonlinear noise regression problem (refer to, e.g., Ormiston et al., 2020; Vajente et al., 2020; Mogushi et al., 2021; Yu et al., 2021). By inputting to a CNN sufficiently many auxiliary witnesses that contain all the information about the noise coupling, and utilizing properly designed network structure and training strategies, we can let the algorithm figure out the coupling mechanism behind the noise even it involves nonlinearity and complicated blending of different sensors. Furthermore, after the training process, the subsequent predicting of the contamination using CNNs is highly efficient computationally, allowing the noise cleaning to be performed in real time (i.e., online). This would be especially beneficial for searches that require low latency, such as the early warning of binary neutron star mergers (Baltus et al., 2021; Yu et al., 2021). Other successful usage of ML techniques in GW astronomy include the identification of various GW events (Bayley et al., 2020; Chan et al., 2020; Dreissigacker and Prix, 2020; Huerta et al., 2020; Krastev, 2020; Schäfer et al., 2020; Wong et al., 2020; Beheshtipour and Papa, 2021; Chang et al., 2021; Chatterjee et al., 2021; López et al., 2021; Marianer et al., 2021; Mishra et al., 2021; Saiz-Pérez et al., 2021; Wei and Huerta, 2021; Yan et al., 2021), source parameter estimations (Gabbard et al., 2019; Chatterjee et al., 2020; Chua and Vallisneri, 2020; Green et al., 2020; Talbot and Thrane, 2020; Álvares et al., 2021; D'Emilio et al., 2021; Krastev et al., 2021; Williams et al., 2021; Xia et al., 2021), and detector characterization (Biswas et al., 2020; Colgan et al., 2020; Cuoco et al., 2020; Essick et al., 2020; Torres-Forné et al., 2020; Mogushi, 2021; Sankarapandian and Kulis, 2021; Soni et al., 2021; Zhan et al., 2021). Besides GW astronomy, the usage of CNNs has led to breakthroughs in a variety of topics related to time-series forecasting and classification (e.g., Refs. Ahmed et al., 2010; Fawaz et al., 2019; Pavlyshenko, 2019; Lim and Zohren, 2021 and references therein), as well as image denoising (e.g., Refs. Pravin and Ojha, 2020; Ilesanmi and Ilesanmi, 2021; Zhou et al., 2021 and references therein).

In this study, we explore in detail how we could use CNNs to potentially mitigate the angular noise in aLIGO, which is the limiting noise source of the current sensitivity in the 30Hz band and is also a classical example of bilinear coupling in aLIGO. Our implementation utilizes the code Keras (Chollet et al., 2015), a deep learning application programming interface written in Python, running on top of the ML platform TensorFlow (Abadi et al., 2015).

The rest of the article is organized as follows. In Section 2, we describe the coupling mechanism behind the angular noise and how we generate mock data so that our study can be carried out in a controlled way. The mathematical details will be supplemented in the Appendix 1. We then explore in Section 3 different CNN structures that can be employed to tackle the nonlinear noise regression problem, with a focus on a general deep-filtering structure and one inspired by the physics of the interferometer. This is followed by Section 4 in which we compare the performance of the two structures. In Section 5, we assess the CNN performance as a function of the signal-to-noise ratio, or SNR, in both the target channel (GW readout) and the input witnesses. We then demonstrate in Section 6 the effects of curriculum learning (CL), which may help the convergence of a CNN when its SNR is low in the target. Finally, we conclude and discuss the implications for future research in Section 7.

## 2. Simulating the Angular Noise in the LIGO System

In this study, we will focus on using CNNs to mitigate noise due to the angular control system, which is one of the major noise sources limiting the sub-30 Hz sensitivity of aLIGO currently (Buikema et al., 2020). To make our study controlled, we will use simulated time series for various channels with characteristics similar to the real aLIGO system. Throughout this study, we will use a fixed sampling rate of 128 Hz for all the time series.

**Figure 1:**
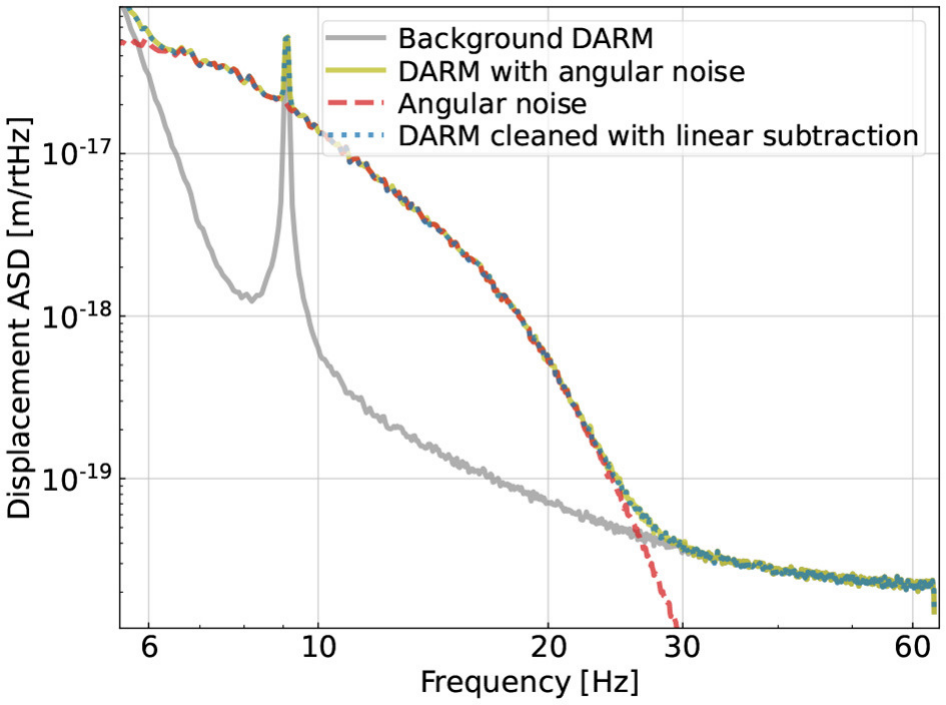
Simulated amplitude spectral density (ASD) of the gravitational-wave (GW) readout(“Differential ARM length (DARM)”) including both a fundamental component (grey trace given by the designed sensitivity; LIGO Scientific Collaboration, 2015) and an extra source of contamination due to the angular noise (Equation A1). The excess noise is above the fundamental limit by about an order of magnitude in the 10-25 Hz band, which is the case for Advanced LIGO (aLIGO) during its third observing run (Buikema et al., 2020). Because the coupling mechanism is bilinear, a standard linear subtraction cannot mitigate the contamination as shown in the dotted-blue trace.

In Figure 1 we show a typical plot of the amplitude spectral density (ASD) of our simulated GW readout including both the fundamental noise (grey trace; which cannot be further reduced by offline regression) and the simulated angular noise (red-dashed trace). Note that the coupling of the angular noise to the main GW readout is bilinear (which we will describe in detail shortly). Consequently, a standard linear regression fails to mitigate its contamination (blue-dotted trace), which thus motivates us to investigate regression strategies utilizing ML. In this Section, we sketch out our simulation of the angular noise (red trace in Figure 1) and present the details in Appendix 1.

At the current power level of aLIGO, the angular control noise couples to the main GW readout (i.e., the monitor of the Differential ARM length, or “DARM”) *via* a geometrical effect. If the beam spot is not at the center of the rotational pivot of an aLIGO test mass (which is also a mirror), then an angular motion will be converted to a length fluctuation bilinearly as Barsotti et al. (2010) and Yu (2019).


(1) $$\begin{array}{r} \delta x^{\left(\text{mir}\right)}\left(t\right)=x_{\text{spot}}^{\left(\text{mir}\right)}\left(t\right)\theta^{\left(\text{mir}\right)}\left(t\right). \end{array}$$

Here, θ^(mir)^(*t*) corresponds to a fast (≳10Hz) angular perturbation to the mirror, which is further induced by the sensing noise in the angular control system being fed back to the mirror. The $x_{\text{spot}}^{\left(\text{mir}\right)}$, on the other hand, corresponds to a slow (≲1Hz) motion of the beam spot on the test mass induced by the seismic motion.

In reality, the mirrors are not controlled locally (in the “mirror basis”) but instead on a radiation-torque basis. This choice is to tackle the Sidles-Sigg effect (Sidles and Sigg, 2006; Hirose et al., 2010; Dooley et al., 2013): as the alignment changes, it creates a radiation torque feeding back to the alignment. In other words, the alignments of the input and end test masses are no longer independent but coupled together *via* the radiation torque. Depending on the sign of the feedback, the radiation torque either hardens (i.e., makes it stiffer) or softens (makes it less stiff) the restoring torque of pendulums suspending the test masses. This allows us to decompose the alignments of the two test masses into a hard mode and a soft mode. The angular control is then performed in terms of this radiation pressure basis.

In aLIGO, the tolerance on the residual hard-mode motion is more stringent, and therefore, it has a greater control bandwidth (with a unity-gain frequency UGF≃3Hz) than the soft mode (with a UGF≲1Hz). As a result, only the hard-mode control feeds back a significant amount of its sensing noise in the 10−30Hz band while the soft mode has negligible motion at high frequency. Consequently, in our simulation, we only simulate θ^(h)^ for the high-frequency angular motion and set θ^(s)^ = 0, where we have used the superscript “h” (“s”) to denote the motion in the hard (soft) mode. Approximately, the spectral shape of θ^(h)^ corresponds to the red trace in Figure 1 (refer to also, Martynov et al., 2016; Yu, 2019; Buikema et al., 2020).

**Figure 2:**
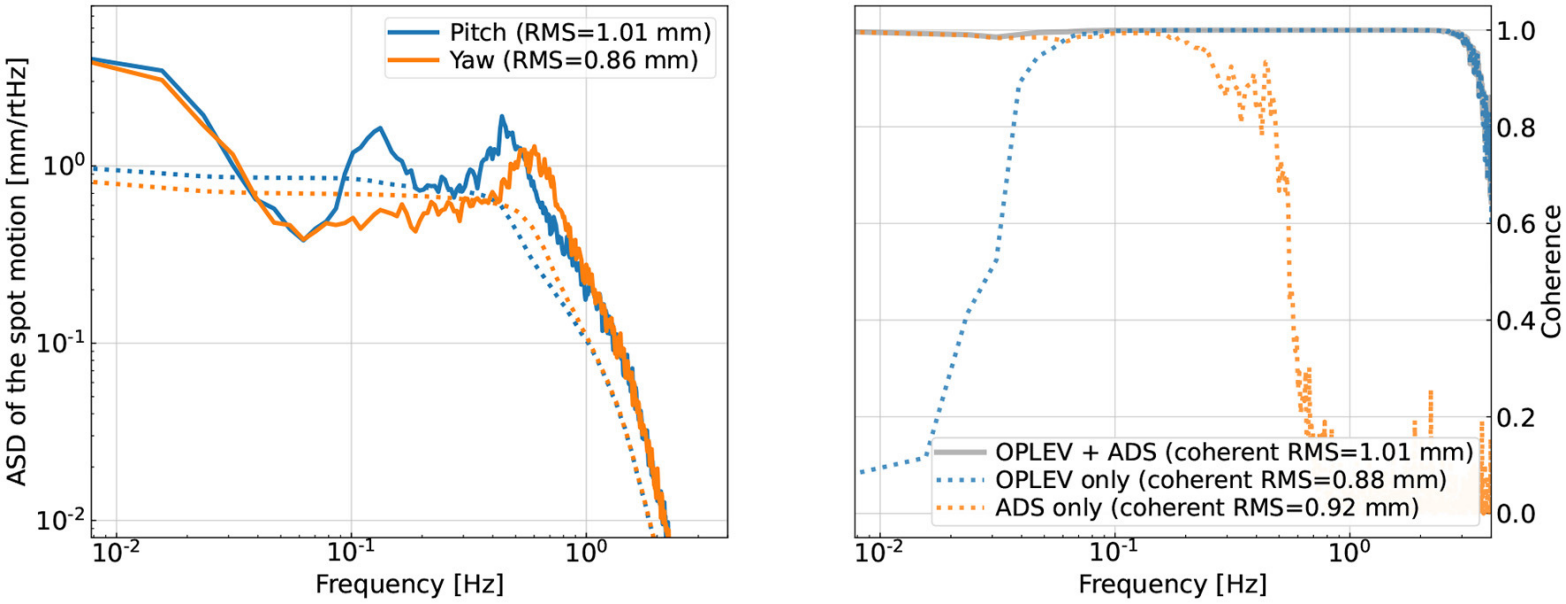
Left panel: ASDs of the simulated beam-spot motions on one of the test masses. Right panel: the coherence between the true spot motion and the simulated witness channels. Here, the dotted lines correspond to the coherence using a single set of witness sensors, and the solid curve corresponds to the multi-input-single-output (MISO) coherence obtained when all the sensors are used. The >0.1Hz motion can be well sensed by a pair of OPLEVs (for the input and end test masses) whose outputs are the angular motion relative to the local chamber. We assume that the <0.1Hz motion is sensed by an alignment dithering system (ADS) channel that senses the spot by measuring the angle-to-length coupling coefficient.

After introducing the high-frequency angular motion θ(*t*), we now describe how we simulate the low-frequency spot motion *x*_spot_(*t*). Here, we simulate directly the motion of each mirror.^1^ For an ideal suspension, the mirror should only have a significant amount of angular motion in pitch because at the suspension point, the seismic motion is mostly longitudinal and the suspension should only couple it into pitch but not yaw. However, in reality, the suspension is not perfectly balanced; the imperfections in control loops also introduce cross-couplings between different degrees of freedom. As a result, the total root-mean-squared (RMS) motions in pitch and yaw can be comparable. Therefore, in our simulation, we assume they have similar RMS values of ≃1mm. We nonetheless give them different spectral shapes as shown in the left panel of Figure 2 as a challenge mimicking the real aLIGO system for our CNN to tackle.

Unlike the high-frequency angular fluctuations that are directly readout in the control sensors, in aLIGO there are no sensors that directly probe the low-frequency spot motions over the entire band of interest. Instead, we would have to mix different sensors' outputs in a frequency-dependent way in order to reconstruct the true spot motion. In fact, the reconstruction of *x*_spot_(*t*) is the most challenging component in mitigating the angular noise in the real aLIGO system currently.

In our simulation, we assume the spot motion can be reconstructed by two sets of sensors. One set senses the spot motion *via* a modulation-demodulation technique. Following LIGO convention, we will refer to these sensors as “ADS” sensors (with ADS standing for the alignment dithering system; Buikema et al., 2020). They probe the very low frequency (<0.1Hz) portion of the spot motion.

The other set of sensors are known as “optical levers,” and we will refer to them as OPLEVs following the LIGO convention (Black et al., 2010). They sense the spot motion in the 0.1−3Hz range. Consequently, we would need to combine both ADS and OPLEV sensors to reconstruct the spot motion, as shown in the right panel of Figure 2 where the coherence between each sensor and the simulated true spot motion is shown. We use the color orange and blue to, respectively, represent the coherence with an ADS sensor and a pair of OPLEVs (as the spot motion on a mirror depends on the angular motion of both mirrors forming the cavity). The multi-input-single-output (MISO) coherence using all the sensors is shown in the grey trace. In the legend, we also quote the RMS value of spot motion that is coherent with the specific set of sensors (the true spot motion has an RMS of 1 mm here). Note that the coherence is related to the SNR at each frequency bin as


(2) $$\begin{array}{r} {\text{SNR}}^{2}=\frac{\text{Coherence}}{1-\text{Coherence}}. \end{array}$$

In total, we would need to include at least 16 sensors (8 ADSs + 8 OPLEVs) in order to sense spot motions on the 4 test masses in both pitch and yaw over the entire band of interest. Together with 4 fast channels corresponding to the 4 hard mode feedback (as there are 2 ARM cavities and each cavity can move in both pitch and yaw), we include 20 auxiliary channels in total in our simulation of the angular noise.

We note that in reality, more channels may be needed for the spot motion. In the right panel of Figure 2, while the simulated OPLEVs have comparable sensitivity to the real ones, we are nonetheless being optimistic about the SNR of the ADS sensors. As we will see later in Section 5.2, without sufficient SNR in the <0.1Hz band the subtraction performance will be significantly limited. Therefore, in order to achieve successful noise regression in reality, we would need either more low-frequency channels with a more complicated CNN structure or more accurate sensing schemes for the ≲0.1Hz spot motion.

## 3. General vs. Specific CNN Structures

Having described our noise simulation, we now discuss the mitigation of the noise using CNNs. In particular, we discuss the process by which we explore different CNN structures in order to optimize the regression performance.

**Figure 3:**
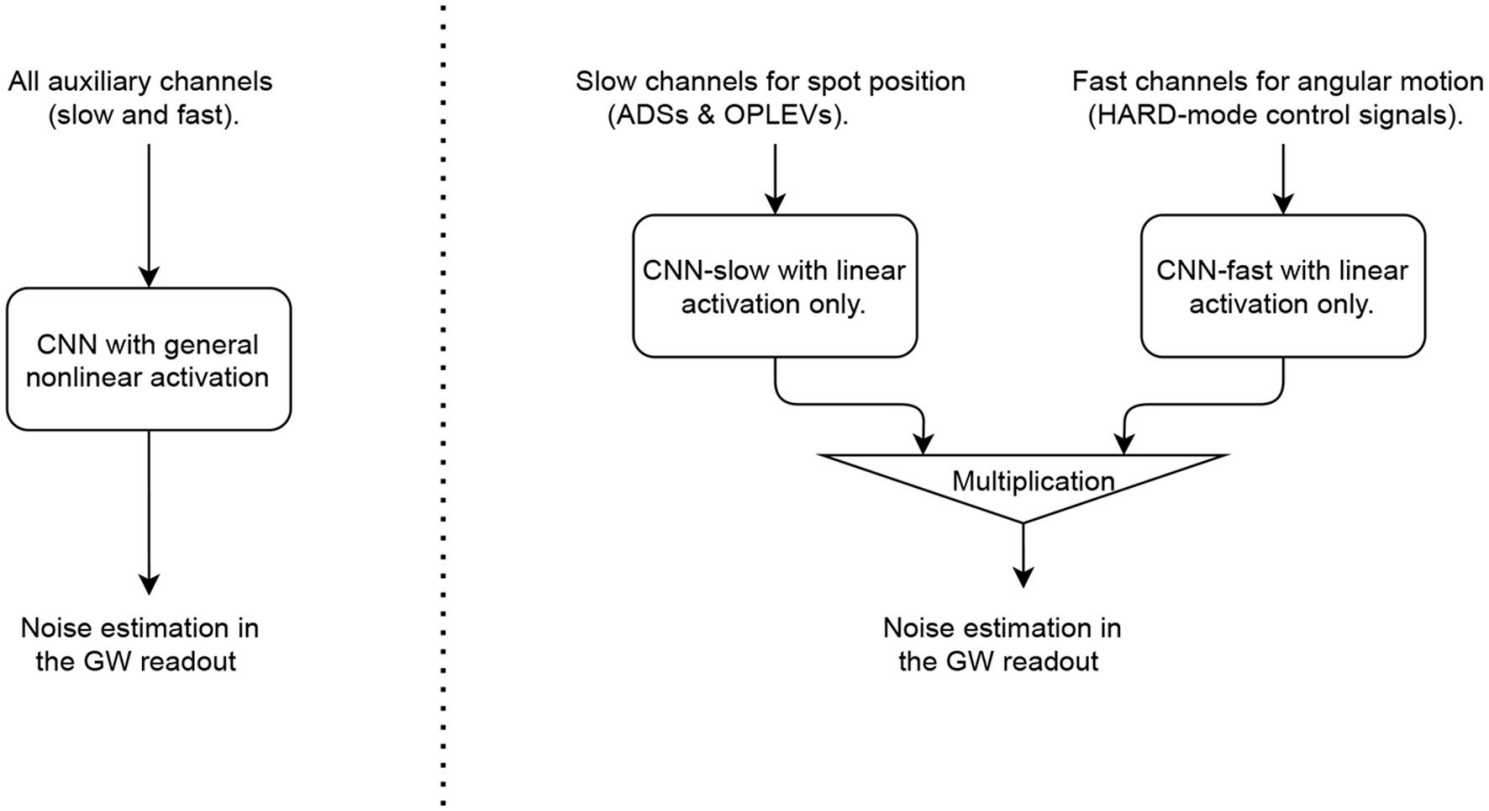
Flow charts of a noise regression convolutional neural network (CNN) utilizing a general structural (left; the parameters are detailed in Table 1) and one that adopts an explicit “slow × fast” structure (right; the parameters are summarized in Table 2).

One option is to use a general deep-filtering structure as illustrated in the left part of Figure 3. Such a CNN should contain sufficiently many convolutional layers together with densely connected layers, and at least some of the layers should involve a nonlinear activation function such as ReLU or ELU at each layer's output. The convolutional layers would then behave similarly to finite-impulse-response filters, enabling the frequency-dependent blending of different auxiliary sensors' time series (i.e., “input” to the CNN). Moreover, with sufficiently many layers with nonlinear activations, the nonlinearity involved in the coupling mechanism can be represented in a series-expansion sense. Such a deep-filtering CNN has the advantage of being straightforward and general. It requires a small amount of prior knowledge and, thus, is particularly suitable to handle noise sources with unknown couplings.

On the other hand, for the bilinearly coupled angular noise described in Section 2, we nonetheless know how the noise propagates to the GW readout. The challenging part of its mitigation is the reconstruction of the spot motion on each test mass. In this scenario, we can in fact utilize our knowledge of the coupling mechanism and design a more specific CNN structure to tackle this problem as shown in the right part of Figure 3. In particular, we divide the auxiliary channels into a slow set [ADSs and OPLEVs for the spot motion *x*_spot_(*t*)] and a fast set [HARD mode control signals for the angular motion θ^(*h*)^(*t*)]. Each set goes through a CNN (which will be referred to as “CNN-slow” and “CNN-fast,” respectively) which requires only linear activation. These CNNs act effectively as FIR filters to convert linearly (yet in frequency-dependent ways) the auxiliary witnesses outputs in digital counts into “super-sensors” that monitor the instantaneous spot positions and alignments of the LIGO test masses. Once the CNNs have constructed the super-sensors, the nonlinearity required by this problem is given by Equation (1) which is made explicit *via* a multiplication layer. For the rest of the Section, we will then demonstrate how the specific structure may improve the mitigation performance relative to the general one. In aLIGO, the coupling mechanisms behind many noise sources are in fact established (refer to, e.g., Martynov et al., 2016 and Buikema et al., 2020 and references therein; refer to also Section 7). This Section, thus, serves further as a demonstration of the benefits of incorporating our knowledge on the instrument when considering noise regression problems.

To compare the performance of the two structures, we consider CNNs with hyper-parameters listed in Tables 1, 2, respectively for the general and the “slow×fast” structures. Note here the input is a 3-dimension array. The first dimension corresponds to the batch dimension (i.e., different realization of the simulated data). The second dimension corresponds to the number of input channels. This is the dimension that is densely connected to allow the CNNs to recognize the correct combinations of different channels as required by, e.g., the underlying cavity geometry. It starts as 20 for the general structure, 16 for CNN-slow, 4 for CNN-fast, and its shape changes according to the “output dimension” column in Tables 1, 2 in subsequent layers. Note that for the final layer it should be 1 corresponding to the main GW readout (i.e., the target of the training). Finally, the final dimension corresponds to the temporal dimension and it is the axis along which the convolution is performed to achieve frequency-dependent filtering.

**Table 1:** Network using a general structure.

	**Layer**	**Output dimension**	**Kernel size**	**Activation**
General structure	Conv1D	32	1,024	Linear
	Dropout	–	–	rate = 8 × 10^−6^
	Conv1D	128	32	Linear
	Conv1D	16	8	Linear
	Conv1D	128	16	Linear
	Dropout	–	–	rate = 8 × 10^−6^
	Dense	128	–	ELU
	Dense	64	–	ELU
	Dense	32	–	ELU
	Dense	8	–	ELU
	Dense	1	–	Linear

*All the 20 auxiliary channels (16 slow channels and 4 fast ones) are directly input to the first layer and the nonlinearity is achieved by a series of Exponential Linear Unit (ELU) activation in the hidden layers. In this network, there are about 8,63,000 trainable parameters in total*.

**Table 2:** Network explicitly utilizing the “slow×fast structure.”

	**Layer**	**Output dimension**	**Kernel size**	**Activation**
slow path	Conv1D	32	1,024	Linear
	Dropout	–	–	rate = 1 ×10^−8^
	Conv1D	128	32	Linear
	Conv1D	16	8	Linear
	Conv1D	128	16	Linear
	Dropout	–	–	rate = 1 × 10^−8^
	Dense	128	–	Linear
	Dense	64	–	Linear
	Dense	32	–	Linear
	Dense	4	–	Linear
fast path	Conv1D	16	8	Linear
	Dense	64	–	Linear
	Dense	32	–	Linear
	Dense	4	–	Linear
after the multiply layer	Dense	32	–	Linear
	Dense	8	–	Linear
	Dense	4	–	Linear
	Dense	1	–	Linear

*We input the 16 slow channels to the slow path and use a series of linear operations (signal filtering and blending) to form 4 super-sensors that effectively monitor the spot position on each test mass. These super-sensors' output is then multiplied with the 4 angular witnesses input to the fast path by a multiply layer to eventually become a length signal. Note that all the activations are linear as the only nonlinearity is explicitly incorporated by the multiplied layer. This network has about 7,36,000 trainable parameters in total*.

In LIGO, the spot position is not directly sensed but it has to be reconstructed from different sets of sensors. Therefore, most of the trainable parameters are in the CNN-slow path which contains 4 convolutional layers followed by 4 densely connected layers. In the first convolutional layer, we further choose a kernel size of 1,024 (corresponding to 8 s of data) such that the CNN can achieve low- and high-passing at ~0.1Hz (Figure 2). Other hyperparameters are tuned empirically at the current stage. It is possible to enhance the network's performance further with more educated and exhaustive tuning, though we defer this optimization to future studies.

The fast angular motions, on the other hand, are directly sensed in aLIGO. Therefore, the CNN-fast path requires only a small number of trainable parameters. In fact, if the fast sensors are properly preconditioned to physical angles θ^(*h*)^(*t*) (by filtering them with the aLIGO suspension model), they can be directly fed to the multiplication layer. Here, we nonetheless leave a small CNN-fast network in the path so that the preconditioning does not need to be exact.

To make fair comparisons, we intentionally keep the CNN using the general structure (Table 1) to be similar to CNN-slow with comparable numbers of trainable parameters. We empirically choose ELU activations in the hidden, densely connected layers to achieve the nonlinear operation in Equation (1).^2^

We employ a custom loss function L based on the band-limited PSD of the GW readout as


(3) $$\begin{array}{r} L=k\int_{f_{1}}^{f_{2}}\frac{S_{n}\left(f\right)}{S_{n}^{\left(0\right)}\left(f\right)}{\left(\frac{f}{f_{1}}\right)}^{\alpha }df, \end{array}$$

where *S*_*n*_ is the PSD of the GW readout (i.e., the target channel) after the subtraction it is normalized by $S_{n}^{\left(0\right)}$, the original PSD before noise subtraction (which is fixed during the training). We have additionally included a power-law weighting with an index α; we empirically set α = −1 in our case. The loss is computed over the frequency band [*f*_1_, *f*_2_], and we tune the overall gain *k* such that the initial loss is about order unity. Note that Equation (3) does not constrain the DC value of the GW readout. While such a DC offset in the GW readout does not directly affect the sensitivity to astrophysical sources, we nonetheless choose to avoid introducing any DC offsets during noise regression. This is achieved by adding the regular mean-square error together with Equation (3) to form our final loss function used during the training process. The relative weights are tuned such that the mean-square error contributes about 10% to the total loss initially.

Because the RMS value of the spot motion contains physical information, we do not normalize each channel by its variance [which may be different for different datasets as the RMS of *x*_spot_(*t*) varies over time]. Instead, we want each channel to be always normalized by the same constant. For this purpose, we calibrate the ADS channels to be in [mm], OPLEVs in [mrad], and the main GW readout (“DARM”) in [fm]. For the HARD-mode error signals, we calibrate them to [pm] and further multiply them by the open-loop gain to precondition them to be proportional to the physical θ^(*h*)^(*t*).

To train the CNNs to recognize the underlying physical coupling mechanism in Equations (1) and (1), we simulate 1,536 s of data for the main GW readout channel (target) and another 20 time series of the same length for the auxiliary witness channels (inputs to the CNNs). Out of the 20 auxiliary channels, 16 are used for the construction of the spot positions, and 4 are used for the fast angular perturbations. All the channels are sampled at the same rate of 128 Hz. During the training, we further divide the time series into 8 batches, each containing 192 s of data. Besides the training data, we additionally prepare 256 s of data for validation and 256 s for testing. We train each CNN until the loss on the validation data plateaus.

## 4. Comparing the Extrapolation Performance

After describing the two network structures in the previous section, we now compare their performance.

**Figure 4:**
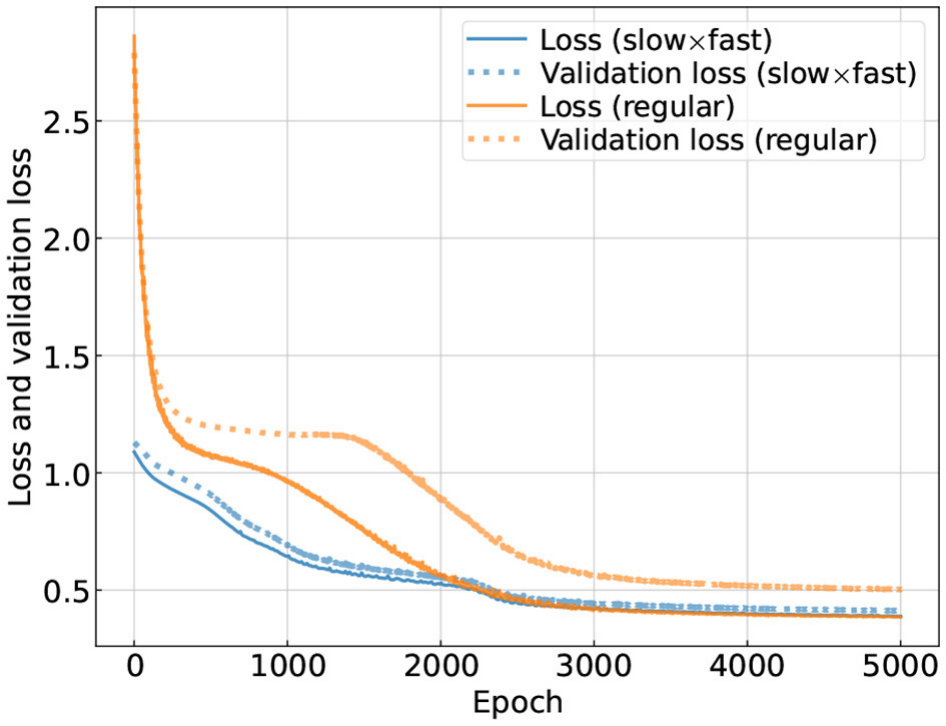
Loss and validation loss [evaluated according to Equation (3)] as functions of the training epoch. We use the colors blue and orange to represent the loss for the CNN using the “slow×fast” structure and the one using the regular structure, respectively. For the test data (corresponding to the left panel of Figure 5), the loss is 0.40 (0.45) for the CNN with the “slow×fast” (regular) structure.

One interesting comparison is the convergence speed which we examine in Figure 4 by showing the loss and the validation loss as functions of the training epoch. Because the “slow×fast” CNN employs our knowledge of the noise coupling mechanism, it naturally converges faster than the regular CNN assuming little prior knowledge. Eventually the training loss for the two CNNs plateaus at similar values, yet for the specific one, the validation loss follows the loss more closely than the regular one, indicating that the specific structure is less subject to over-fitting.

**Figure 5:**
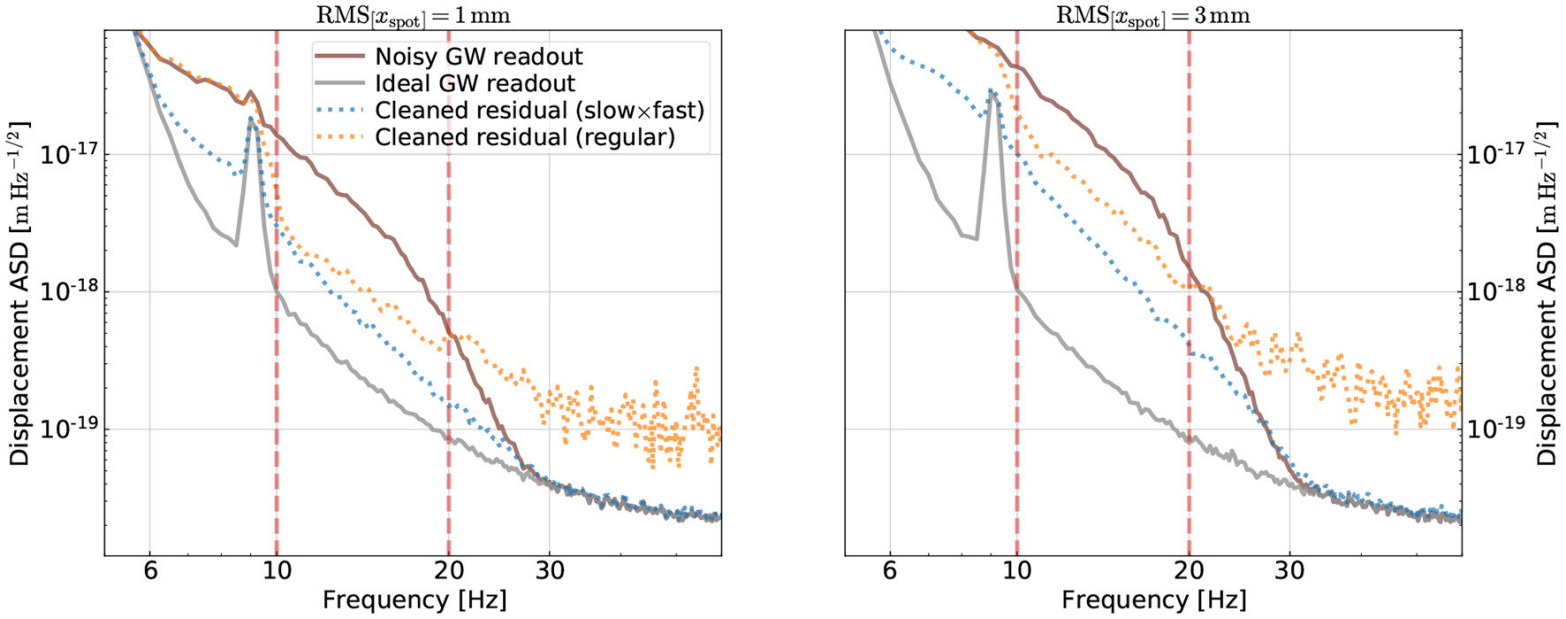
Comparison of the noise subtraction results using the “slow×fast” structure vs. the general structure with the ELU activation. In the left, the spot motion has an RMS value of 1mm, which is the same as the training data set. The two vertical lines indicate the band used for computing the loss (i.e., the training band). Using the “slow×fast” structure, we not only achieve a better subtraction in the training band but also avoid injecting extra contamination outside the band. In the right panel, we extrapolate the results on testing data with spot motion 3 times higher than the one used for training. The performance of the “slow×fast” degrades less compared to the general one.

More importantly, we can examine the subtraction results on the testing data in Figure 5. Here, the brown trace is the ASD including both the fundamental noise and the bilinear noise. The ASD of the fundamental noise is also shown separately in the grey trace; in the ideal case, a CNN should use the information stored in the auxiliary channels to reduce the brown ASD to the grey one. The ASDs of residual time series cleaned with our CNNs are shown in the dotted traces. Here, we use the color orange and blue to respectively represent the results obtained from the general (Table 1) and the “slow×fast” (Table 2) CNNs.

One question we want to address is how well each CNN extrapolates. Particularly, one quantity we extrapolate is frequency. While during the training we would like to focus on the frequency band where the noise contamination is the highest to get the best mitigation there, we also want to avoid injecting excess noise outside this band during the subtraction process. To test this point, we, thus, set the training band (i.e., the band where we compute the loss function in Equation 3) to be [*f*_1_, *f*_2_] = [10, 20]Hz, corresponding to the two red-vertical lines in Figure 5. As shown in the figure, inside the [10, 20]Hz band both the general and the “slow×fast” CNNs have decent and comparable subtraction performance (about a factor of 10 at 10 Hz; the “slow×fast” CNN has a slightly better performance at other frequencies). The losses are 0.45 and 0.40 for the general and the “slow×fast” CNNs, respectively. Outside the training band, on the other hand, the “slow×fast” CNN significantly outperforms the general one. Whereas, the general CNN starts to inject excess noise into the GW readout almost immediately outside the training band, the one adopting the “slow×fast” structure continues to reduce the noise for the entire band from 6 Hz to 30 Hz where the angular noise is above the fundamental limit. Above 30 Hz, the “slow×fast” CNN also avoids making the noise worse than the fundamental limit. We, thus, see that by utilizing our knowledge of physical systems, we can achieve good extrapolation properties with respect to frequency.

Meanwhile, we would also like to assess how the CNNs perform at different values of RMS of the spot motion. This is because in the real aLIGO system the spot motion is induced by the <1Hz seismic motion which varies over time, and the CNN's performance would, thus, need to be robust against the variation in the spot motion. We address this point by simulating an additional 256 s of data with an RMS spot motion of 3 mm and then testing our CNNs on this dataset with 3 times higher spot motion than the training dataset. The result is summarized in the right panel in Figure 5. While the fractional noise reduction at 10 Hz degrades for both CNNs, the degradation is less if one uses the “slow×fast” structure than the general structure.

## 5. SNR Requirements

In the section above, we have considered a simple case where the total GW readout contains only a fundamental component and all the excess contamination is due to the angular noise. In reality, there are many other types of excess noise in the low-frequency part of the aLIGO sensitivity band whose information is not captured in the auxiliary channels we input to the CNN. Effectively, the presence of other contamination reduces the angular noise's SNR in the GW readout (i.e., the target channel; note, here, we treat the angular noise as the signal because it is what we want to remove by the CNN). We, thus, explore how the CNN's performance varies with respect to the GW readout in Section 5.1.

Another thing we would like to explore is the SNR of ADS sensors for the very low frequency (≲0.1Hz) spot motion. The coherence level shown in the right panel of Figure 2 is likely to be an optimistic estimation for the ≲0.1Hz band because sensing the spot motion in this band is intrinsically challenging. Not only do the ADS sensors have high sensing noise as described in Section 2, but also the spot estimation could be biased by other contamination mechanisms.^3^ Therefore, we also assess the CNN's performance with respect to the SNR in the ADS sensors in Section 5.2.

### 5.1. In the GW Readout

To modify the SNR of the simulated angular noise in the main GW readout, we simulate the total length fluctuation δ*x*_tot_ in the GW readout as


(4) $$\begin{array}{r} \delta x_{\text{tot}}\left(t\right)=\delta x_{\text{fun}}\left(t\right)+\delta x_{\text{ang}}\left(t\right)+\delta x_{\text{oth}}\left(t\right), \end{array}$$

where δ*x*_fun_ corresponds to the fundamental noise component (grey trace in Figure 1), δ*x*_ang_ is the angular noise we want to subtract remove (red trace in Figure 1), and δ*x*_oth_ are other types of contamination to DARM whose information is not contained in the auxiliary channels we input. For the other contamination, we further assume that it has an ASD given by $\sqrt{S_{\text{oth}}\left(f\right)}\propto f^{-3}$. This way, we can control the SNR[=$\sqrt{S_{\text{ang}}\left(f\right)}/\sqrt{S_{\text{fun}}\left(f\right)+S_{\text{oth}}\left(f\right)}$] of the bilinear noise by changing the overall magnitude of the other noise's ASD.

**Figure 6:**
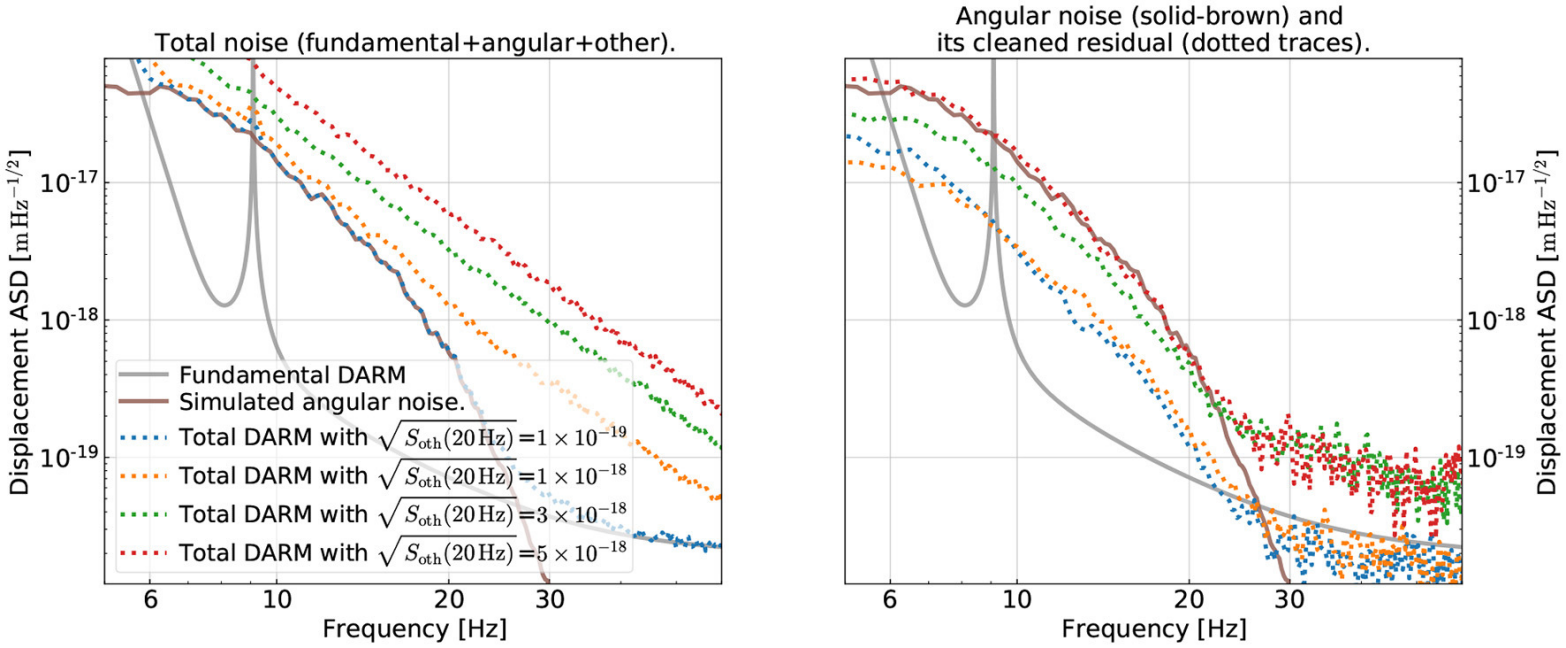
Examining the subtraction performance at different levels of signal-to-noise ratio (SNR) in the GW readout (i.e., “DARM”). Here, “SNR” means the ratio of the angular noise to the other, non-angular noises in DARM (including the fundamental noise and other technical noises whose summed ASD is denoted by $\sqrt{S_{\text{oth}}}$). In the left panel, we show the DARM spectra at different values of $\sqrt{S_{\text{oth}}}$ (dotted lines), while holding the angular noise fixed at the brown-solid trace. In the right panel, we show the subtraction residual of the angular noise (the lower the better). The color coding of each dotted trace is the same as in the right panel. We see that the subtraction has similar performance when the SNR≳1 (blue and orange traces), while it degrades quickly when SNR < 1 (green and red traces).

In the left panel of Figure 6, we show the ASDs of the total DARM displacement, now including the contribution from other contamination simulated according to Equation (4). We use dotted lines with different colors to indicate different levels of the other noise while the angular noise is held fixed as shown in the solid-brown trace in this section. For each level of SNR, we regenerate 2,048 s of data (1,536 s for training, 256 s for validation, and 256 second for testing). We then train our CNN on the new data and check how much it could mitigate the bilinearly coupled angular noise (solid-brown trace in the left panel of Figure 6). Here, we focus on the CNN with the “slow×fast” structure (Table 2) as it has a better performance than the general one (Section 3). The auxiliary channels are still assumed to have good sensitivity as shown in the right panel of Figure 2. The training strategy follows the one outlined in Section 3 except for that we now evaluate the loss, Equation (3), over a broader band of [*f*_1_, *f*_2_] = [8, 40]Hz.

In the right panel, we show the ASDs of the residual time series after noise mitigation by our CNN. For presentation purposes, we show specifically the residual angular noise component in each dotted line. This is obtained by subtracting from the residual [i.e., the difference between the original δ*x*_tot_(*t*) and the one predicted by our CNN] further the δ*x*_fun_(*t*) and δ*x*_oth_(*t*) components when generating the plot, which is possible as we are dealing with the simulated data. Different colors correspond to different levels of SNR and the correspondence is the same as in the left panel.

As shown in the plot, when the angular noise has an SNR≳1 at each frequency bin in the GW readout, the CNN can achieve decent and consistent subtraction performance as shown by the blue and orange traces. At 10 Hz, the angular noise could be reduced by about an order of magnitude. However, when the SNR drops below unity as shown in the green trace (with an SNR of about 0.5 at 10 Hz), the CNN could only marginally reduce the angular noise in the 10-20 Hz band, and it starts to inject excess contamination at higher frequencies.

Therefore, for the CNN to be able to recognize the correct noise coupling mechanism, it would typically need an SNR of unity in the target channel. Nonetheless, we note that it is not a *necessary* condition. As we will discuss later in Section 6, we may still be able to remove contaminations in DARM with a sub-unity SNR if we utilize active injection and CL.

### 5.2. In the Witness Sensors

So far we have assumed the ADS sensors have good SNR of the true spot motion below 0.1 Hz as indicated in the left panel of Figure 2. This may be an optimistic assumption in reality as discussed at the beginning of Section 5. We, thus, explore here how does the SNR in the ADS channels (i.e., inputs or witnesses) affects the performance of the CNN.

**Figure 7:**
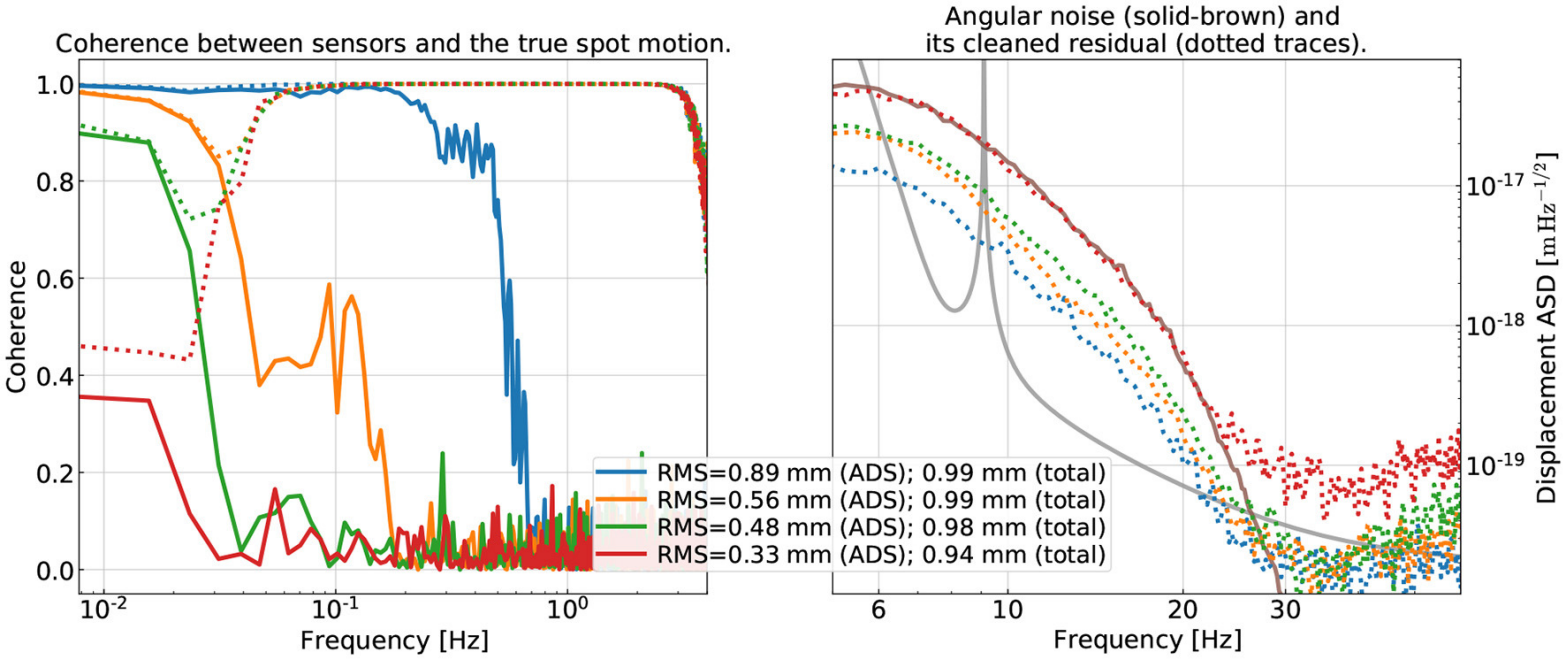
Examining the subtraction performance at different levels of SNR in the low-frequency ADS channels (which probes the true beam spot motion ≲0.1Hz). In the left, we show the coherence between an ADS channel (solid traces) and the true spot motion on one of the test masses in pitch. As a reference, the total MISO coherence using two OPLEVs and one ADS as the witnesses is shown in the dotted traces. In the legend, we show the RMS of the spot motion coherent with the witnesses (either a single ADS or further combined with two OPLEV sensors; the total spot RMS is 1 mm in all cases). On the right, we show the subtraction residual of the angular noise with the color of each dotted trace corresponding to the same level of sensor SNR as in the left. While we can reconstruct ≳95% in the power of the true spot motion even in the red traces (the worst case considered), the subtraction degrades significantly as the SNR in the ADS channels reduce. It, thus, indicates the necessity of having good sensors covering the entire frequency band of interests.

For this purpose, we vary the sensing noise in each ADS channel to modify its coherence with the true spot motion. This is indicated in the left panel of Figure 7. We use the solid trace to represent the coherence between an ADS's output and the true spot motion it senses, and the dotted trace the MISO coherence if we further include a pair of OPLEVs. In the legend, we quote the RMS value of the spot motion that is coherent with the sensors (the true spot motion has an RMS of 1mm). Note the coherence is related to the SNR *via* Equation (2).

We repeat the training process now on data with noisy ADS sensors and the subtraction result is summarized in the right panel of Figure 7. The color coding of each curve has the same meaning as in the left panel. We see that as the ADS sensors' sensitivity decreases, the noise subtraction performance degrades significantly. Take the green trace as an example. Even the MISO coherence is greater than at least 0.7 over the entire band of interest and the RMS of the spot motion coherent with the sensors (ADS + OPLEVs) almost matches the RMS of the true value, the amount of noise the CNN can subtract reduces by a factor of 2 compared to the blue trace. Our study, thus, suggests that the high-accuracy reconstruction of the beam spot on each test mass over the entire <1Hz band is crucial for the success of the mitigation of the angular noise. In fact, spot reconstruction should be a topic deserving dedicated studies in its own right.

## 6. Curriculum Learning

As discussed in the previous section, in order for the CNN to be able to remove the angular noise, we would need to have good SNRs in both the target (the main GW readout) and the witness sensors (the ADS sensors). On the other hand, the difference exists between the two scenarios. If the SNR is low in the witnesses (Section 5.2), it means that we do not have enough information to recover the noise coupling and the only solution is to incorporate more sensors to recover the missing information. If the SNR is low only in the target (Section 5.1), then the fact that the CNN does not achieve a good subtraction is simply due to it not converging to the right physics during the training process. All the necessary information to predict the angular noise is still available. In this case, we show in this section that we can still have decent mitigation if we utilize CL techniques (George and Huerta, 2018a,b).

The key idea behind CL is the following. We can first train the CNN on datasets with a high SNR in the target channel, which can be produced in reality with active injections. A high-SNR dataset is a simple task to be tackled and it helps the CNN to converge to the right physics initially. We can then gradually increase the task's difficulty by incorporating data with lower excitation levels, and eventually the quiet-time data (i.e., data without any active injections). Since the CNN is guided through the training process, it stays close to the true physics and can thus predict the desired target even using quite-time data. This process is especially suitable to be combined with a physically-inspired CNN structure (such as the “slow×fast” one) that has a good extrapolation property.

We demonstrate this point, here, by first training our CNN on data corresponding to the blue traces in Figure 6.^4^ After its convergence, we then incorporate into the training set the data corresponding to the orange traces (as different “batches”) and continue training the CNN obtained from the first step until its validation loss plateaus. In the last step, we further include data corresponding to the red trace (with the lowest SNR in the GW readout) and retrain the CNN from the previous step to convergence.

**Figure 8:**
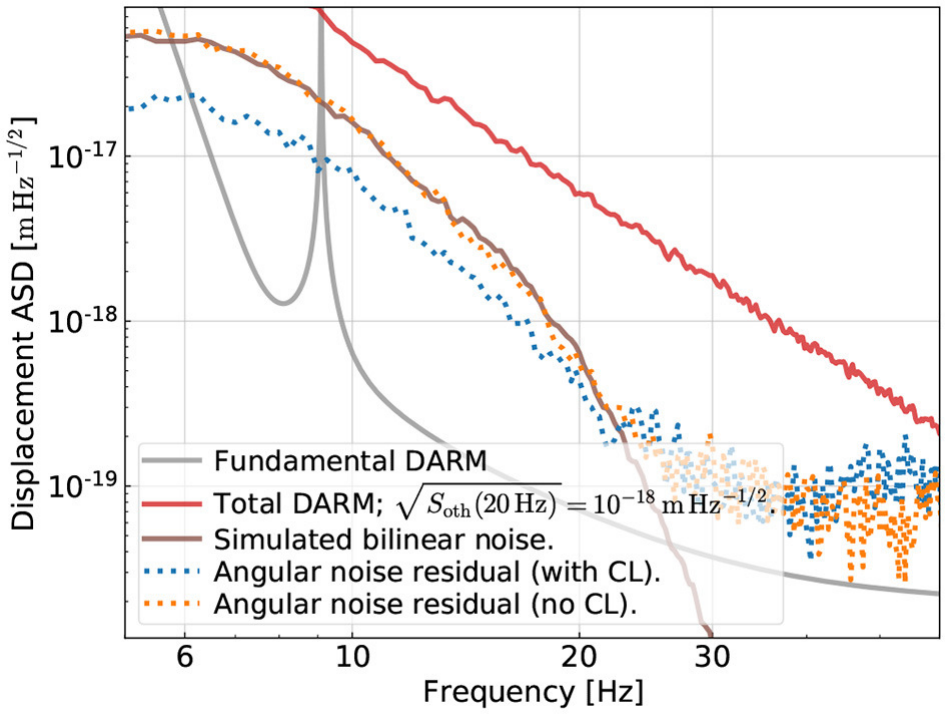
Examining the subtraction performance with or without curriculum learning (CL). Here, we consider the same dataset as shown in the red traces in Figure 6. That is, while the angular noise (subtraction target) is high compared to the fundamental DARM noise, it is buried by other technical noises (i.e., the SNR of the angular noise is less than unity). Without CL, no mitigation of the angular noise is achieved (as in Figure 6). Using CL, on the other hand, allows the network to achieve better performance. This suggests that in reality, we can use active noise excitation and CL training to enhance the performance of noise subtraction on the quiet-time data.

After the CL training, we test the resultant CNN on the 256-s testing data for the red trace in Figure 6 and the result is presented in Figure 8 in the dotted-blue trace. As a comparison, we also showed the subtraction result without CL in the dotted-orange trace (using the CNN obtained in Section 5.1). Whereas the CNN could not mitigate any of the angular noise without CL, we see that with the help of CL the CNN can reduce the noise by a factor of 2 at around 10 Hz. It, thus, demonstrates that CL could be a useful training strategy to help the CNN remove noise that has a low SNR in the GW readout (yet it can still be high compared to the fundamental limit and, thus, needs to be tackled).

## 7. Summary and Discussion

In this study, we explored how we may use CNNs to improve the sub-60 Hz sensitivity of aLIGO. Here, we focused specifically on the bilinearly-coupled angular noise which is one of the limiting noise sources in the 10-30 Hz band. Using simulated data with characteristics similar to the real aLIGO system (Section 2), we explored various factors affecting a CNN's performance. One of the most critical factors is to utilize our knowledge in the design of CNN structures (Section 3). Specifically, a “slow×fast” structure is suitable to mitigate the angular noise as it incorporates the nonlinearity involved in the problem in an explicit way, which leads to good extrapolation properties (Section 4). We further explored the SNR requirements in both the target (GW readout; Section 5.1) and the witness sensors (ADS sensors; Section 5.2) for the CNN to converge. To overcome the lack of SNR in the witnesses, it would require improving the sensing technology and/or including more sensors to recover the information. On the other hand, when the SNR is only low in the target, we demonstrated in Section 6 that CL training can be used to facilitate the CNN's convergence.

While in this study, we focused on the angular noise that couples to DARM according to Equation (1), we note that the coupling mechanisms of many other noises in LIGO share the same bilinear structure and can be modeled as a fast channel modulated by a slow one (i.e., the product of the two). For example, the signal-recycling cavity's length fluctuation (a fast channel) couples to DARM with a slow modulation due to low-frequency variations in the DARM offset. Other noise like light scattering, when expanded into a Taylor series, will also have terms similar to Equation (1) serving as the lowest-order nonlinear terms. Therefore, the “slow×fast” CNN structure we present in Section 3 will have broad applications in LIGO noise mitigation beyond just the angular noise. We plan to explore this point more in future studies.

We note that the CL result shown in Figure 8, while having an improved performance compared to the case without CL, has not yet reached a level comparable to the case where it converges to the right physics (e.g., the blue and orange traces in the right panel of Figure 6). It, thus, indicates that the detailed CL steps have rooms for further optimization, which we defer to be explored by future studies.

Throughout the analysis, we have focused on using simulated data. As we have mentioned in the main text, mitigating the angular noise in the real aLIGO system is more challenging. This is because there are many other noise sources in the same frequency band (including ones that are not yet captured by the current noise budgeting; Martynov et al., 2016; Buikema et al., 2020), limiting the SNR of the angular noise in the GW readout. Moreover, to reconstruct *x*_spot_(*t*), it is likely requiring more sensors than just the ADSs and OPLEVs. We also ignored potential transients (“glitches”) in both the GW readout and witness sensors, which is another crucial application of ML in GW astrophysics in its own right (refer to, e.g., Cuoco et al. (2020) and references therein). On the real data, our CNN has not yet achieved a significant broadband reduction of nonlinearly coupled noises, yet it shows promising signs such as removing sidebands around dithering lines that are used to create the ADS signals (Yu and Adhikari, 2021). We plan to investigate this further in future studies, especially combining it with a fine-tuned CNN hyper-parameter set and an optimized CL training strategy. Meanwhile, LIGO has released a 3-h data stretch in its second observing run including major auxiliary channels^5^. We would, thus, like to encourage interested readers to use either the CNN structures we proposed in this study or original CNN structures to help the further improvements of aLIGO's sensitivity.

## Data Availability Statement

The raw data supporting the conclusions of this article will be made available by the authors, without undue reservation.

## Author Contributions

HY performed the training and testing of the CNNs. RA supervised the research. Both authors contributed to the article and approved the submitted version.

## Funding

HY is supported by the Sherman Fairchild Foundation. RA is supported by NSF grant no. PHY-1764464. The authors gratefully acknowledge the computational resources provided by the LIGO Laboratory and supported by NSF grant nos. PHY-0757058 and PHY-0823459.

## Conflict of Interest

The authors declare that the research was conducted in the absence of any commercial or financial relationships that could be construed as a potential conflict of interest.

## Publisher's Note

All claims expressed in this article are solely those of the authors and do not necessarily represent those of their affiliated organizations, or those of the publisher, the editors and the reviewers. Any product that may be evaluated in this article, or claim that may be made by its manufacturer, is not guaranteed or endorsed by the publisher.

## Footnotes

^1^Because the hard-mode and soft-mode control loops have different shapes, the spot motion in the hard mode and the soft mode in principle will have different values (similar to the case of high-frequency angular motion). However, subtleties arise because the spot motion governing the length fluctuation in Equation (1) is measured with respect to a local rotational pivot, whereas the angular control is performed relative to an input beam (i.e., a global quantity). At low-frequencies (<1Hz), the two reference points drift independently. Our current simulation does not capture this effect and for simplicity, we treat the spot motion on each mirror as an independent.

^2^In our case, we empirically find that having nonlinear activations in the convolutional layers does not significantly enhance the subtraction performance but makes the convergence slower. Therefore, we apply only the linear activation to the convolutional layers.

^3^For example, the power circulating in the arm cavity may be modulated by θ^(mir)^(*t*) due to both the residual angular motion at <1Hz and/or imperfections in the mirror coating. The power fluctuation could further lead to a longitudinal perturbation mitigated by radiation pressure in addition to the geometrical effect Equation (1). Thus, the effective spot position inferred at a given dithering frequency could be a biased estimation for another frequency.

^4^In reality, $\sqrt{S_{\text{oth}}}$ is approximately fixed and to create high-SNR data we should instead inject excess motion to, e.g., θ^(*h*)^. Nonetheless, since what matters most is the SNR of the bilinear noise in the GW readout, here, we simply reuse the existing data.

^5^The data can be downloaded at https://www.gw-openscience.org/auxiliary/GW170814/.

## A. Details of Noise Simulation

In this Appendix, we supplement details of our simulation of the angular noise presented in Section 2.

Note that Equation (1) describes the local length fluctuation of a single mirror [and hence a superscript “(mir)” is added to each quantity]. A physical interest is the length fluctuation of an ARM cavity formed by an input test mass and an end test mass. In other words, we want to measure the relative distance between the two test masses (which are also mirrors) as the GW signal is readout *via* the differential-ARM (DARM) length. Therefore, the angular noise affects the cavity length as

(A1)
$$\begin{array}{r} \delta x\left(t\right)=\left[\begin{array}{cc} x_{\text{spot}}^{\left(\text{e}\right)}\left(t\right) & x_{\text{spot}}^{\left(\text{i}\right)}\left(t\right) \end{array}\right]\left[\begin{array}{c} \theta^{\left(\text{e}\right)}\left(t\right)\\ \theta^{\left(\text{i}\right)}\left(t\right) \end{array}\right], \end{array}$$

where the superscript “(e)” [“(i)”] stands for the end (input) test mass. Its contamination to DARM is further obtained by first incoherently simulating each cavity's length according to Equation (1) and then taking the difference.

As we described in Section 2, the real angular control is performed on a radiation pressure basis (Sidles and Sigg, 2006; Yu, 2019), which can be understood as the following. Mathematically, we note that the radiation torque depends on the spot motions on the test masses, which further relate to the alignment of the test masses *via* the cavity geometry. For pitch motion (yaw motion is similar), this is given by Sidles and Sigg (2006), Barsotti et al. (2010), and Yu (2019).

(A2)
$$\begin{array}{r} \left[\begin{array}{c} x_{\text{spot}}^{\left(\text{e}\right)}\\ x_{\text{spot}}^{\left(\text{i}\right)} \end{array}\right]=\frac{L}{g^{\left(\text{e}\right)}g^{\left(\text{i}\right)}-1}\left[\begin{array}{cc} g^{\left(\text{i}\right)} & 1\\ 1 & g^{\left(\text{e}\right)} \end{array}\right]\left[\begin{array}{c} \theta^{\left(\text{e}\right)}\\ \theta^{\left(\text{i}\right)} \end{array}\right]\equiv M_{\text{a}2\text{s}}\left[\begin{array}{c} \theta^{\left(\text{e}\right)}\\ \theta^{\left(\text{i}\right)} \end{array}\right], \end{array}$$

where *L* is the length of the ARM cavity, and *g*^(e)^ = 1−*L*/*R*^(e)^ [and similarly *g*^(i)^ = 1−*L*/*R*^(i)^] with *R*^(e)^ [*R*^(i)^] the radius of curvature of the end (input) test mass. For aLIGO, we have *L* = 3, 995m, *g*^(e)^ = −0.779, and *g*^(i)^ = −1.065 (Barsotti et al., 2010). The hard (soft) mode then corresponds to the eigenvector of the ***M***_a2s_ matrix associated with a positive (negative) eigenvalue. The conversion between the mirror basis ([θ^(e)^, θ^(i)^]*T*) and the radiation-torque basis ([θ^(h)^, θ^(s)^]*T*) is given by

(A3)
$$\begin{array}{r} \left[\begin{array}{c} \theta^{\left(\text{h}\right)}\\ \theta^{\left(\text{s}\right)} \end{array}\right]=M_{\text{m}2\text{r}}\left[\begin{array}{c} \theta^{\left(\text{e}\right)}\\ \theta^{\left(\text{i}\right)} \end{array}\right]=\left[\begin{array}{cc} 0.756 & -0.655\\ 0.655 & 0.756 \end{array}\right]\left[\begin{array}{c} \theta^{\left(\text{e}\right)}\\ \theta^{\left(\text{i}\right)} \end{array}\right], \end{array}$$

where in the second equality, we have plugged in numerical values for the aLIGO interferometer. In our simulation, we set θ^(s)^ = 0 and simulate only θ^(h)^ as only the hard mode has a significant control bandwidth and feeds back its sensing noise in the 10–30 Hz band. Above the UGF, the physical θ^(*h*)^ is the product between a white sensing noise with a typical ASD of ~5 × 10^−14^radHz^−1/2^ and the open-loop gain of the angular control loop (Martynov et al., 2016; Yu, 2019; Buikema et al., 2020). Once we have θ^(h)^, it can then be converted back to the mirror basis by inverting Equation (3).

To sense the low-frequency spot position motion *x*_spot_(*t*), we simulate two sets of sensors similar to the realistic aLIGO system. One set corresponds to the ADS sensors. Specifically, we intentionally excite each mirror in angle at a known frequency and then demodulate the length readout (i.e., DARM output) at the same frequency. From Equation (1) we see that the demodulated signal is directly proportional to the spot motion on the test mass. However, this technique has a limited SNR because only a weak excitation is allowed in order to avoid saturation of actuators and sensors as well as up-conversion of the low-frequncy noise into the sensitivity (>10Hz) band. Consequently, the ADS sensors are sensitive to only the very low frequency (<0.1Hz) portion of the spot motion.

To probe the spot position in the 0.1-3 Hz band, we employ the OPLEV sensors. An OPLEV probes a mirror's alignment locally, which then allows us to infer the spot motion using Equation (2). However, the reference point drifts slowly, and thus, an OPLEV can only probe spot motion in the ≳0.1Hz band. One of the main challenges to the CNN in our study is, thus, to recognize the frequency-dependent blending of the ADS and OPLEV sensors according to the right panel of Figure 2.

Finally, we point out that the channels we simulate are in fact based on real LIGO auxiliary channels. The fast angular motion θ^(h)^(*t*) are contained in channels like L1:ASC-CHARD_P_IN1_DQ. The slow channels are designed to mimic auxiliary channels like L1:ASC-ADS_PIT4_DOF_OUT_DQ for the ADS sensors, and L1:SUS-ITMX_L3_OPLEV_PIT_OUT_DQ for the OPLEVs.
